# Genetic mapping of yield traits using RIL population derived from Fuchuan Dahuasheng and ICG6375 of peanut (*Arachis hypogaea* L.)

**DOI:** 10.1007/s11032-016-0587-3

**Published:** 2017-01-30

**Authors:** Yuning Chen, Xiaoping Ren, Yanli Zheng, Xiaojing Zhou, Li Huang, Liying Yan, Yongqing Jiao, Weigang Chen, Shunmou Huang, Liyun Wan, Yong Lei, Boshou Liao, Dongxin Huai, Wenhui Wei, Huifang Jiang

**Affiliations:** Oil Crop Research Institute, Chinese Academy of Agricultural Sciences/Key Laboratory of Biology and Genetic Improvement of Oil Crops, Ministry of Agricultural, Wuhan, 430062 People’s Republic of China

**Keywords:** Peanut (*Arachis hypogaea* L), QTL analysis, Yield traits, Seed length, Seed width, Pod weight, Seed weight

## Abstract

The genetic architecture determinants of yield traits in peanut (*Arachis hypogaea* L.) are poorly understood. In the present study, an effort was made to map quantitative trait loci (QTLs) for yield traits using recombinant inbred lines (RIL). A genetic linkage map was constructed containing 609 loci, covering a total of 1557.48 cM with an average distance of 2.56 cM between adjacent markers. The present map exhibited good collinearity with the physical map of diploid species of *Arachis*. Ninety-two repeatable QTLs were identified for 11 traits including height of main stem, total branching number, and nine pod- and seed-related traits. Of the 92 QTLs, 15 QTLs were expressed across three environments and 65 QTLs were newly identified. Twelve QTLs for the height of main stem and the pod- and seed-related traits explaining more than 10 % of phenotypic variation showed a great potential for marker-assisted selection in improving these traits. The trait-by-trait meta-analysis revealed 33 consensus QTLs. The consensus QTLs and other QTLs were further integrated into 29 pleiotropic unique QTLs with the confidence interval of 1.86 cM on average. The significant co-localization of QTLs was consistent with the significant phenotypic correlations among these traits. The complexity of the genetic architecture of yield traits was demonstrated. The present QTLs for pod- and seed-related traits could be the most fundamental genetic factors contributing to the yield traits in peanut. The results provide a good foundation for fine mapping, cloning and designing molecular breeding of favorable genes in peanut.

**Electronic supplementary material:**

The online version of this article (doi:10.1007/s11032-016-0587-3) contains supplementary material, which is available to authorized users.

## Introduction

The cultivated peanut (*Arachis hypogaea* L.) is a major oil and food crop in most tropical and subtropical areas of the world. The major objectives are to increase grain yield and improve the selection efficiency in peanut breeding. The yield traits, such as the height of main stem (HMS), total branching number (TBN), and the pod and seed traits directly contributed to grain yield in peanut (Holbrook and Stalker 2003; Shirasawa et al. 2012). The 100 pod weight (100PW), 100 seed weight (100SW), and shelling percentage (SP) are important index for grain yield of peanut. They were determined by pod and seed size, which were quantified by the pod length (PL), pod width (PW), pod thickness, seed length (SL), seed width (SW), and seed thickness (Shirasawa et al. 2012). The pod and seed traits are the most directly selected during long-term domestication and breeding. Research on them is also helpful to understand the yield component and evolution of crop species (Moles et al. 2005; Meyer and Purugganan 2013).

Marker-assisted selection (MAS) is a powerful tool for enhancing selection efficiency. The basis of MAS is the construction of genetic map and the identification of the major genes or markers which are directly associated with the objective traits (Knapp 1998; Francia et al. 2005). Enough simple sequence repeat (SSR) markers have been developed in peanut research community for its virtues of transferable nature and the practical handle, and the construction of integrated consensus map also allowed a transverse comparison between populations (Gautami et al. 2012; Qin et al. 2012; Shirasawa et al. 2013; Huang et al. 2016; Zhou et al. 2016). The remarkable progress had been made on construction of genetic map and quantitative traits loci (QTLs) detection for important traits in peanut (Burow et al. 2001; Garcia et al. 2005; Gomez Selvaraj et al. 2009; Hong et al. 2010; Gautami et al. 2012; Qin et al. 2012; Shirasawa et al. 2012; Wang et al. 2012a, b, 2015a; Jiang et al. 2014; Pandey et al. 2014; Zhou et al. 2014; Huang et al. 2015, 2016). MAS had been used successfully in quality improvement, nematode, and rust resistance breeding in peanut (Chu et al. 2009, 2011; Sujay et al. 2012; Varshney et al. 2014; Wang et al. 2015a; Janila et al. 2016). Markers had also been developed for drought tolerance in peanut (Gautami et al. 2012).

Up to date, the MAS for peanut yield breeding is still in progress. For the HMS and TBN, a number of QTLs were identified (Fonceka et al. 2012; Shirasawa et al. 2012; Jiang et al. 2014; Huang et al. 2015). QTLs for the pod and seed traits were also identified using linkage and association mapping. Eighteen QTLs for PL and 24 QTLs for PW were identified, which explained 1.24–28.2 and 1.13–22.3 % of phenotypic variation, respectively (Gomez Selvaraj et al. 2009; Fonceka et al. 2012; Shirasawa et al. 2012; Jiang et al. 2014; Huang et al. 2015; Chen et al. 2016a, b). Eighteen QTLs for SL and 15 QTLs for SW were located on different linkage groups, which explained 9.86–16.3 and 6.39–23.7 % of phenotypic variation, respectively (Gomez Selvaraj et al. 2009; Huang et al. 2015; Chen et al. 2016a, b). There were also 13 and 5 QTLs for SL and SW, respectively, which were identified using association analysis (Jiang et al. 2014; Pandey et al. 2014). Up to date, as an important index of exterior quality, the study on the ratio of PL to PW and SL to SW was not reported. For 100PW, six QTLs explaining 1.69–20.6 % of phenotypic variation were located on linkage A2, A8, B2, B3, and B5 linkage groups (Fonceka et al. 2012; Huang et al. 2015). The minor QTLs with 1.22–2.26 % of phenotypic variation explained were also detected using association analysis (Jiang et al. 2014). For 100SW, six QTLs with 8.02–19.1 % of phenotypic variation explained were located on five linkage groups (Gomez Selvaraj et al. 2009; Fonceka et al. 2012; Shirasawa et al. 2012; Huang et al. 2015), and five QTLs with 12.73–26.08 % of phenotypic variation explained were identified using association analysis (Pandey et al. 2014).

The genetic basis of seed size and weight were also characterized well in other crops and the pleiotropy for these traits was observed. In soybean, the QTLs for seed size and weight were distributed over 16 chromosomes and many of them explained minor or moderate phenotypic variation (Salas et al. 2006; Xu et al. 2011; Niu et al. 2013; Kato et al. 2014). The QTL X40, X53, X83–X85, and X92 simultaneously controlled two of the SL, SW, and 100SW (Xie et al. 2014). In rice, the genes controlling seed-related traits acted in independent genetic pathways. The *GS3*, *GS5*, *qGL3/qGL3.1*, *GW5/qSW5*, and *TGW6* simultaneously affected two or more seed size traits and grain quality, indicating a complex genetic basis of these traits (Zuo and Li 2014; Peng et al. 2015; Wang et al. 2015b). The tight linkage and pleiotropy were observed on the silique length, seed size, and weight in *Brassica napus*. The gene *ARF18* simultaneously affected seed weight and silique length (Liu et al. 2015). Three major QTLs simultaneously controlled seed weight and silique length (Yang et al. 2012; Li et al. 2015). However, compared to the comprehensive QTLs research on rice, soybean, and oilseed rape, it is still far from comprehensively understanding the genetic factors controlling these traits in peanut (Varshney et al. 2010, 2013; Pandey et al. 2014).

Recently, the genome sequences of the two diploid species of *Arachis* were published (Bertioli et al. 2016; Chen et al. 2016a, b) which are the common ancestors of cultivated peanut. It would be greatly facilitated for genetic map validation and QTLs mapping. The main objectives of the present study were (1) construction of a genetic map using recombinant inbred lines (RIL) population; (2) mapping of QTLs for yield traits including the HMS, TBN, pod- and seed-related traits; and (3) comparative mapping of QTLs between the physical and the genetic maps. These results would provide comprehensive information for QTL comparison among similar studies of peanut, MAS of yield breeding, and map-based cloning of the candidate genes in peanut.

## Materials and methods

### Plant material

A RIL population including 188 lines was developed by single seed descent from a cross between female parent Fuchuan Dahuasheng (*A. hypogaea* L. subsp. *hypogaea* L. var. *hirsuta* Kohle) and male parent ICG6375(*A. hypogaea* L. subsp. *fastigata* Waldron var. *vulgaris* Harz) (Fig. S1). Fuchuan Dahuasheng is a local cultivar in Guangxi Province of southwest China, ICG6375 is introduced from International Crops Research Institute for the Semi-arid Tropics (Fig. S1). This combination was chosen because of their big differences in major agronomic traits including the pod’s shape and size; seeds’ shape, size, and coat color; flowering time, number of seeds per pod (50 % pods of Fuchuan Dahuasheng were three-seeded); and total branching number. The phenotypic value of traits in the present study was listed in Table S1.

### DNA extraction

The young unopened leaves were collected from each accession for genomic DNA extraction using a modified CTAB method. Total DNA was digested with RNase and then quantified with a Beckman DU-650 spectrophotometer. Agarose gel electrophoresis was employed for evaluation of quality and integrity. The PCR product were visualized by 6 % denaturing polyacrylamide gel (PAGE) followed by silver staining as described by Chen (2008).

### Field trials and trait phenotyping

The population comprising 188 lines was grown in 2013 (*F*_5_) and 2014 (*F*_6_) seasons. Ten individuals of each line were evaluated for the HMS, TBN, PL, PW, PL/PW, SL, SW, SL/SW, 100PW, 100SW, and SP. Phenotyping of *F*_5_ RIL population was carried in Wuchang (environment 1, E 114° 34′/N 30° 59′) and Yangluo (environment 2, E 114° 52′/N 30° 59′) in 2013, and phenotyping of *F*_6_ RIL population was carried in Wuchang in 2014 (Environment 3, E 114° 34′/N 30° 59′) for two trials. The two-seeded pods and seeds were measured by their length and width using a parallel rule. The pods and seeds weight were measured by an electrical scale. The PL/PW and SL/SW were calculated using PL and PW and SL and SW. The SP was calculated as 100PW divided by the seed weight of 100 pods.

### Statistical analysis

Phenotypic data were tested for normality using the PROC UNIVARIATE procedure of SAS 9.3 (SAS Institute, Cary, NY, USA). Correlation coefficients among 11 traits were estimated using the PROC CORR procedure of SAS. The broad-sense heritability for each trait was calculated by a method described by Wu et al. (2009).

### SSR markers analysis

A total of 8456 SSR primer pairs were employed to detect their polymorphism between parents of RIL population. The selected polymorphic markers were assigned to genotype the RIL mapping population. PCR was performed in a total volume of 10 μl of reaction mixture consisted of 0.4 mM dNTPs, 2.0 mM Mg^2+^, 1× Taq buffer, 0.5 U Taq DNA polymerase (Tiangen), 0.3 μM primers, and 25 ng genomic DNA. Amplification was carried out on a T100™ Thermo cycler (BIO-RAD). The parameters of thermal cycle were 95 °C for 3 min, followed by 10 cycles of 94 °C for 30 s, 65 °C for 30 s (decreasing in decrements of 0.5 °C per cycle), and 72 °C extension for 60 s; then followed by another 30 cycles of 94 °C for 30 s, 55 °C for 35 s, 72 °C for 60 s, and final 72 °C for 5 min.

### Construction of genetic map and comparison between the genetic and physical maps

Genotyping data obtained from the RIL population was used for linkage analysis using JoinMap 4.0 (Van Oojen and Voorips 2006). Segregation distortion of each marker was examined at an expected 1:1 using chi-squared (*χ* ^2^) testing under “Locus genotype frequency” function. The “Kosambi mapping” function (Kosambi 1944) was used to transform recombination frequencies into centiMorgan (cM) map distances with a minimum log-of-odds (LOD) threshold of 4.0 and recombination frequency of 0.35. Marker order in groups was corrected under the “Calculate Map” command. The comparison between the present genetic map and the physical map was based on the markers assigned to the chromosomes of A genome of *Arachis duranensis* and B genome of *Arachis ipaensis* (Bertioli et al. 2016, http://peanutbase.org/download). The corresponding genomic position of markers on the present genetic map was determined using blast/e-PCR with the primer sequence against the physical map of *A. duranensis* and *A. ipaensis*. The comparison between the present genetic map and the integrated consensus map (the INT map, Shirasawa et al. 2013) was based on the position of common markers. The compared map was generated using MapChart for Windows (Voorrips 2002).

### QTLs mapping

The respective data of single environment and the averaged data of each 2013 and 2014 were combined with marker genotyping data to identify QTLs, respectively. The average value of phenotyping data across three environments was also employed to detect the QTLs which were expressed across the three environments. QTL analysis was conducted by composite interval mapping (CIM) using Windows QTL Cartographer 2.5 (Wang et al. 2012a, b). The LOD value was chosen to be 2.9 to declare a QTL significant based on a permutation test (Churchill et al. 1994) with 1000 runs to determine the *P* = 0.05 genome-wide significance level. To evaluate more potential QTLs, the QTLs with a LOD value more than 2.5 were also referenced. Based on the QTL detection above using Windows QTL Cartographer 2.5, algorithms for QTL meta-analysis were used to estimate the number and position of QTLs which were located at same region on the linkage group (Goffinet and Gerber 2000). This approach provides a modified *Akaike* criterion that can be used to determine the number of meta-QTL that best fitted the results on a given linkage group. The two-round strategy of QTL meta-analysis was performed to integrate the QTLs using BioMeractor2.1 software (Arcade et al. 2004; Chardon et al. 2004). The BioMeractor2.1 set a 95 % confidence intervals for the meta-QTL using the formula $\mathrm{C}.\mathrm{I}.=\frac{3.92}{\sqrt{\sum_{i=1}^{i= k}\Big(1/{S}_i^2}\Big)}$ , where ${S}_i^2$ presents the variance of the position of QTLi, and *k* presents the total number of QTL integrated into the meta-QTLs. Firstly, the additive QTLs expressed in different environments for the same trait were integrated into the consensus QTL. Secondly, the overlapped consensus QTLs and the other QTLs for different traits were further integrated into the unique QTL. The QTL nomenclature was referenced to the research reported by Shi et al. (2009). For the consensus QTL, the QTL nomenclature corresponded to *q* (abbreviation of QTL), followed by the abbreviation of the trait (e.g., HMS); then, the linkage group number; and finally, the serial number. For the unique QTL, the nomenclature corresponded to *uq*, followed by the linkage group number, and then the serial number.

## Results

### Phenotypes of mapping population

**Fig. 1:**
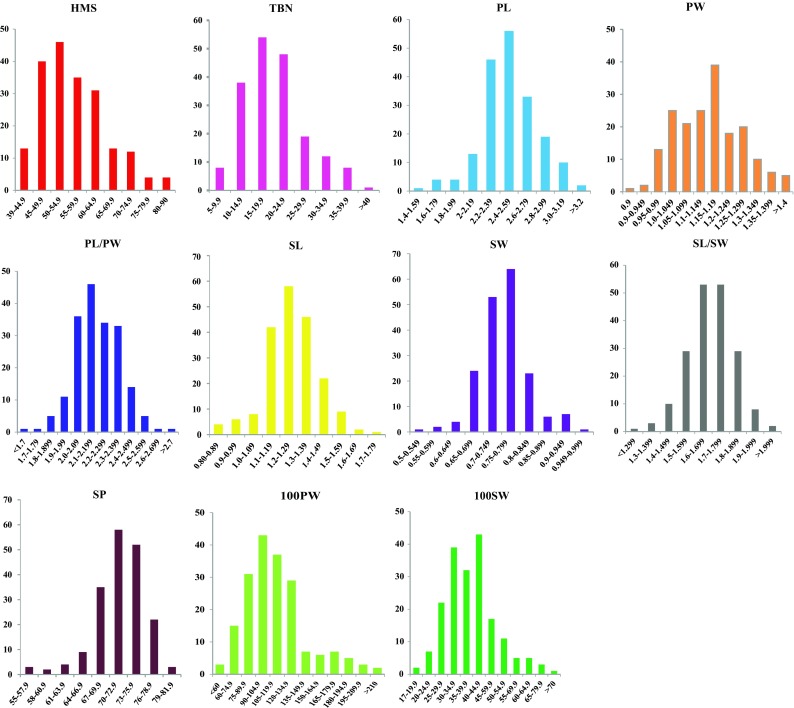
Phenotypic distribution of 11 yield traits in the RIL population

The population was phenotyped extensively for 11 traits. For all of the traits except SW and SP, significant differences were observed between Fuchuan Dahuasheng and ICG6375 (Table S1). The average values of all traits across three environments were used in subsequent variance analysis. All traits showed continuous normal or near-normal distribution, indicating a polygenic inheritance of them and an ideal model for QTL analysis (Fig. 1). All traits showed high broad sense heritability ranged from 0.75 to 0.95 except HMS (0.40) and SP (0.59), and SW showed the highest heritability. The correlation coefficient analysis discovered a significant correlation among most of the traits (Table 1). The PL, PW, SL, and SW showed significant positive correlation (*P* < 0.01) with 100PW and 100SW, and PL showed significant negative correlation (*P* < 0.01) with SP. The HMS and TBN were relatively independent of other traits.

**Table 1:** Correlation analysis of the 11 traits in present population across three environments

Correlation	HMS	TBN	PL	PW	PL/PW	SL	SW	SL/SW	100PW	100SW	SP
HMS		*	ns	ns	ns	ns	ns	ns	ns	ns	ns
TBN	0.180		ns	ns	ns	ns	ns	ns	ns	ns	ns
PL	0.004	−0.088		**	**	**	**	**	**	**	**
PW	0.046	−0.130	0.725		**	**	**	**	**	**	**
PL/PW	−0.058	0.109	0.463	−0.267		**	**	**	ns	ns	ns
SL	−0.011	−0.085	0.856	0.733	0.247		**	**	**	**	ns
SW	−0.053	−0.081	0.482	0.820	−0.376	0.604		**	**	**	ns
SL/SW	0.043	0.065	0.559	0.084	0.670	0.620	−0.246		**	**	ns
100PW	0.002	−0.057	0.828	0.881	0.017	0.837	0.786	0.234		**	**
100SW	−0.022	−0.105	0.721	0.863	−0.101	0.851	0.857	0.329	0.914		ns
SP	−0.106	−0.104	−0.259	−0.224	−0.063	−0.136	−0.019	−0.046	−0.326	−0.087	

*ns* non-significant at *P* < 0.05

*Significant at *P* < 0.05;**Significant at *P* < 0.01

### Characteristics of genetic map

**Fig. 2:**
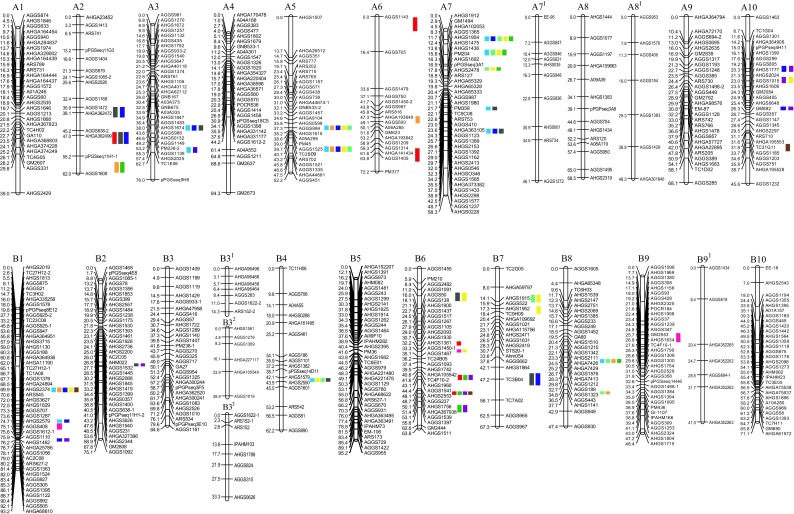
Genetic linkage map and QTL detection of the 11 traits in an RIL population derived from Fuchuan Dahuasheng × ICG6375

A total of 718 of 8456 SSR markers were polymorphic between Fuchuan Dahuasheng and ICG6375. Under the threshold of more than 6 loci for grouping, 609 loci were mapped onto the 26 linkage groups with an average of 23.4 loci per group (Fig. 2, File S1). The map covered a total of 1557.48-cM genetic distance ranging from 14.25 to 95.24 cM with averaged 59.90 cM for each group and 2.56 cM between adjacent markers (Fig. 2). A total of 321/609 (52.71 %) loci showed distorted segregation ration (*P* < 0.01), 238/321 (74.14 %), and 83/321 (25.86 %) loci skewed toward female and male parents, respectively. The groups were designated as A1–A10 and B1–B10 for A and B genome by assigning common markers to the INT map (Table S2, File S2, Figs. S2 and 4). Especially, more than one group shared the same designation and were marked using superscript number when they were assigned to the same one group on INT map. As a result, the short group A7^1^ was attached to A7 group, B3^1^, B3^2^, and B3^3^ were attached to B3 group.

### Homologous relationships of FI map to the physical map and the INT map

**Fig. 3:**
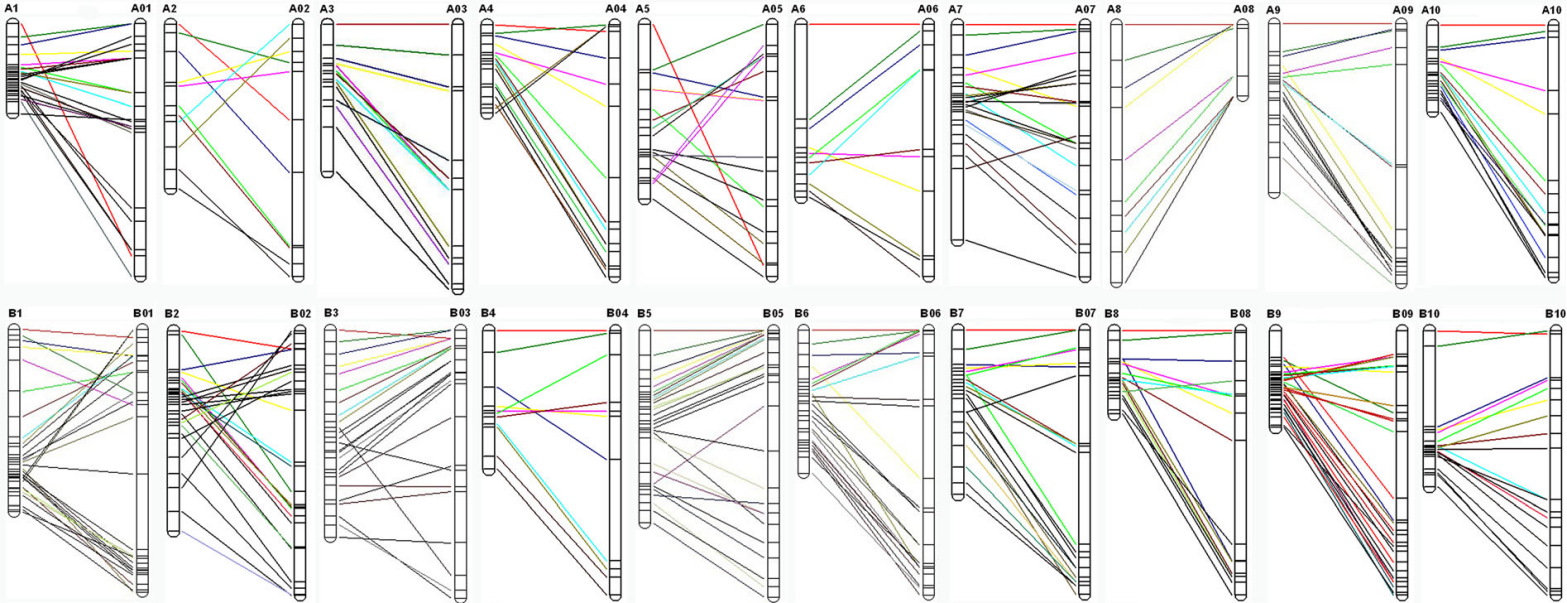
Comparison of the present map and the physical map of A and B genomes of diploid species *A. duranensis* and *A. ipaensis*. Collinear SSR loci between linkage groups of present map and the counterpart chromosomes of A and B genome were indicated by lines

The availability of the genome sequence of diploid species of *Arachis* is helpful to compare the present genetic map and the physical map in *A. hypogaea* (Bertioli et al. 2016, http://peanutbase.org/download). A total of 428 loci presenting 1432.86 cM were successfully aligned to the chromosomes and covered 2007.86 Mb with an average of 1.4 Mb/cM. There were 175 loci representing 626.43 cM and 253 loci representing 806.43 cM were assigned to A and B genomes, and covered 818.0 and 1189.86 Mb, respectively, (Table 2, File S3 and 4). The high collinearity was also observed between the present and the physical maps (Fig. 3, Fig. S3, File S3 and 4). Generally, the good collinearity was observed between A3 and A03, A8 and A08, A9 and A09, B5 and B05, B8 and B08, B9 and B09, and B10 and B10 (Fig. 3, Fig. S4).

**Table 2:** Summary statistical of the present linkage map

Linkage group	Markers number	Genetic distance	Marker density^a^	Anchored markers number^b^	Recombination frequency^c^	Coverage ration^d^	Number of marker distorted to		
							Fuchuang	ICG6375	Total
A1	30	39.02	1.3	24	2.83	0.79	2	16	18
A2	15	62.02	4.43	11	1.37	0.85	3	3	6
A3	31	75.96	2.53	14	1.80	0.83	4	5	9
A4	29	84.3	3.01	16	2.18	0.66	8	1	9
A5	28	52.19	1.93	18	1.44	0.68	0	12	12
A6	17	72.2	4.51	11	1.47	0.80	4	6	10
A7	38	58.25	1.57	23	1.17	0.85	16	2	18
A7^1^	10	46.14	5.13	9	0.06	0.04	3	0	3
A8	14	68.51	5.27	10	0.88	0.37	7	1	8
A8^1^	7	48.27	8.04	4	0.16	0.16	1	1	2
A9	28	68.13	2.52	18	1.53	0.87	14	0	14
A10	29	45.63	1.63	17	2.68	0.85	13	0	13
B1	47	93.2	1.98	30	1.44	0.95	14	5	19
B2	36	75.12	2.15	28	1.32	0.91	16	5	21
B3	30	84.61	2.92	25	1.29	0.80	26	0	26
B3^1^	7	14.25	2.38	5	0.29	0.02	1	2	3
B3^2^	6	39.92	7.98	5	0.10	0.03	0	0	0
B3^3^	8	33.34	4.76	6	0.09	0.02	4	0	4
B4	16	62.21	12.44	11	1.90	0.88	2	4	6
B5	36	95.24	2.72	28	1.38	0.88	20	0	20
B6	31	63.82	2.13	24	1.84	0.84	0	12	12
B7	20	67.32	3.54	24	1.60	0.85	12	2	14
B8	27	47.37	1.82	17	3.14	0.88	5	6	11
B9	35	48.4	1.42	29	2.71	0.89	33	0	33
B9^1^	7	40.96	6.83	6	0.06	0.01	1	0	1
B10	30	71.1	2.45	20	1.69	0.88	29	0	29
Total	612	93.2	2.13	437	1.40	0.65	238	83	321

^a^Marker density is the ration of markers number to genetic distance

^b^Anchor markers number represents the number of markers which was anchored to the sequenced A genome of *A.duranensis* and B genome of *A.ipaensis*

^c^Recombination frequency is the ratio of physical distance to the genetic distance

^d^Coverage ration is calculated as the covered physical distance (Mb) of linkage group divided by the length (Mb) of the corresponding chromosome

For a huge and poorly characterized genome of allotetraploid *A. hypogaea*, it would be helpful to well understand the genetic make-up of tetraploid cultivars of peanut by comparison between the present map and the INT map which showed the highest marker density and the largest genetic distance (Shirasawa et al. 2013). The good agreement of collinear was observed between the present map and the INT map (Fig. S1, File S2). Twenty-four groups including the attached groups of A7, A8, and B3 were homologous to the 20 groups on the INT map. For the A2 group, only one marker was assigned to A02 group of the INT map. A total of 167 loci covered 904.63/714.96 cM in the present map and the INT map, and the collinear region was narrowed in the INT map. Ten groups of 388.0 cM with 72 loci were assigned to A genome, and 12 groups of 516.63 cM with 95 loci were assigned to B genome (Fig. S2 and 4, Table S2, File S2). The fine collinearity was observed between A1 and A01, A7 and A07, A10 and A10, B2 and B02, and B6 and B06 (Figs. S2 and 4).

### QTLs detection and meta-analysis

For all 11 traits, a total of 175 QTLs were detected (File S5). And 92 of them were defined as repeatable QTLs for they were detected in more than two environments or using average data or their overlapped confidence interval for different traits (File S6). The identified QTLs explained 5.1–13.2 % of the phenotypic variation. Eleven QTLs were identified as major QTLs with more than 10 % of phenotypic variation explained, and 15/92 QTLs were also meanwhile detected across three environments (File S6). Where the confidence interval of the QTLs for the same trait in different environments overlapped, meta-analysis was employed to integrate these QTLs into consensus QTL. As a result of the first round of meta-analysis, the 70 QTLs were integrated into 33 consensus QTLs (Table. S3, File S7). For example, eight QTLs for SL were integrated into three consensus QTLs on A7 group (Fig. S5). In the second round of meta-analysis, besides the consensus QTLs, a total of 53 single QTLs for different traits detected were also employed because of their contribution to the correlated traits and the same or very close position on the linkage group. Finally, the 30/33 consensus QTLs and the 53 QTLs were further integrated into 29 unique QTLs (Table S3, File S8). For example of the integration of unique QTLs *uqA7–1*, the five QTLs including three single QTLs *qPLA7.1a*, *qPL/PWA7.1a*, and *q100SWA7.1a*, and two consensus QTLs *qSLA7.1a* and *q100PWA7.1a*, were further integrated into the *uqA7–1* (Fig. S5, Table S3). A total of ten unique QTLs were integrated by the QTLs detected only once, and the remaining 19 unique QTLs were integrated by both the consensus and the single QTLs (Table S3). The average confidence interval of all unique QTLs was reduced from 3.03 to 1.86 cM, and the average genetic distance of linked marker to the peak position was 0.45 cM. A total of 29 markers linked to the unique QTLs were also identified (File S9). The QTLs were mainly located on A2, A3, A5, A6, A7, A10, B1, B2, B4, B6, B7, and B8 groups (Fig. 2).

As a result of the two rounds of meta-analysis, the significant co-localization of QTLs was observed especially for pod and seed traits (Fig. 2, Table S3). A total of 83 QTLs were co-localized at 29 pleiotropic loci which were distributed on the 11 linkage groups. And 13 of 29 unique QTLs controlled at least three different traits. For example, the *uqA5.1a*, *uqA5.1b*, *uqA7.1a*, and *uqB1.1a* represented QTL for five traits. The co-localization of these QTLs was in accordance with the signs of the genetic-correlation coefficients of pod- and seed-related traits.

The HMS and TBN are the most important plant architecture traits. For the HMS, a total of six QTLs were detected repeatedly. And four consensus QTLs were detected and explained 5.95–9.14 % of phenotypic variation. Among them, one was on A6 linkage group and three were on B6 linkage group (Fig. 2, Table S4). QTL *qHMSA6.1a*, *qHMSA6.1b*, *qHMSB6.1a*, and *qHMSB6.1b* were detected across three environments. All four QTLs showed negative additive effect (Table S4). For the TBN, three QTLs, *qTBNB1.1a*, *qTBNB6.1b*, and *qTBNB9.1a* were detected using pooled data of E1 and E2, explaining 5.08–6.03 % of phenotypic variation (Fig. 2). The *qTBNB1.1a* and *qTBNB9.1a* showed positive additive effects of 2.07 and 2.50, while the *qTBNB6.1b* showed negative additive effect of 2.20 (File S5).

In the present study, 83 of 92 QTLs were detected repeatedly for pod- and seed-related traits, and 11 of them were also detected across three environments. Finally, 30/33 consensus QTLs and all 29 unique QTLs were all accounted to these traits (Table S3, Table S4). Almost all the QTLs for pod- and seed-related traits were involved into the generation of unique QTLs, except the *qPWA6.1a*, *qSWA8.1a*, and *qSPA10.1a* (Fig. 2, Table S3). For the PL, 12 QTLs explained 5.6–13.2 % of phenotypic variation, the *q*PLA7.1a was detected across three environments, and four consensus QTLs were located on A5 and A7 groups. Seven QTLs explained 5.6–8.6 % of phenotypic variation of PW, the *qPWB8.1a* was detected across the three environments, and two consensus QTLs were located on B8 group. Thirteen QTLs explained 5.4–12.1 % of phenotypic variation of PL/PW, only one consensus QTLs *qPL/PWB7.1a* was located on the B7 group. Twelve QTLs explained 5.44–13.2 % of phenotypic variation of SL. And as many as seven consensus QTLs for SL were generated, and the consensus QTL *qSLA7.1b* was integrated by four QTLs. Ten QTLs explained 6.0–12.8 % of phenotypic variation of SW, three of which were consensus QTL and located on A10 and B2 groups. For the SL/SW, nine QTLs explained 5.5–11.8 % of phenotypic variation. Of them, the *qSL/SWA2.1a* and *qSL/SWA3.1a* were expressed across three environments, and the *qSL/SWA7.1a* and *qSL/SWA7.1b* were consensus QTL. Interestingly, unlike the *qPLB1.1a*, *qPWB1.1a*, and *qPL/PWB1.1a* were detected independently at the same one locus, although the *qPLB8.1a* and *qPWB8.1a* were detected at the same one locus on B8 group; however, the QTL for PL/PW was not detected on B8 group.

The 100PW, 100SW, and SP are the most direct yield traits. For the 100PW, eight QTLs explained 5.3–11.2 % of phenotypic variation. QTLs *q100PWA7.1a* and *q100PWB7.1a* were detected across three environments, the *q100PWA5.1a*, *q100PWA7.1a*, and *q100PWB8.1a* were consensus QTL. For the 100SW, nine QTLs explained 5.2–10.8 % of phenotypic variation, three of which were consensus QTL. The QTLs *q100SWA7.1a*, *q100PWA7.1b*, *q100PWB6.1a*, and *q100PWB8.1a* were detected across three environments. Three QTLs explained 5.6–10.9 % of phenotypic variation of SP, and the *qSPA10.1a* and *qSPB6.1a* were consensus QTL.

## Map-based comparison of the present and previous QTLs for yield traits

The availability of the common markers enabled the comparison of the present and previous QTLs for yield traits. By common marker and the position of the QTL confidence interval, the present QTLs *qSWA3.1a*, *qPWA5.1b q100PWA7*.*1a*, and *q100PWA7*.*1b* were identical to the *qSWA3*, *qPWA5*, *qHPWA7.1* and *qHPWA7.2* (Huang et al. 2015). One QTL for seed weight identified previously (Pandey et al. 2014) was identical to the present *qPWA2.1a* and *q100SWA2.1a.* The QTL *qPLA5.1a* was identical to the QTL for pod maturity (Gomez Selvaraj et al. 2009). One QTL for the HMS on A6 identified by Shirasawa et al. (2013) was also possible identical to the one of present QTLs for the HMS. And seven QTLs identified by Fonceka et al. (2012) were possibly identical to the present QTLs on A7, A10, and B6 groups. A total of the present 12 QTLs were also detected using *F*_2:3_ populations of the present RIL population (Chen et al. 2016a, b). Totally, there were 27 previous QTLs were reproduced in the present study, and 65 QTLs were newly identified in the present study, and 56 of them were for pod and seed traits which directly contributed to peanut grain yield.

## The comparative mapping of QTLs on the physical map

Due to the good collinearity between the present map and their counterpart chromosomes of the physical map of diploid species of *Arachis*, an attempt was made to evaluate the physical distance of QTLs based on the e-PCR with the present primer sequence against the physical map of *A. duranensis* and *A. ipaensis* (Bertioli et al. 2016). The physical distance of the interval of flanking markers and the confidence interval of QTLs were evaluated based on the position of markers linked to the QTLs on the physical map. A total of 11 unique QTLs with flanking markers were directly assigned to the physical map (File S9). The physical distance ranged from 0.71 to 28.2 Mb with an averaged distance of 9.82 Mb. Interestingly, the *uqA7–1* and *uqA7–2* were located in the region of 1.29 and 0.71 Mb. For tracking the result in further study, the physical position of the markers linked to the peak position was also listed (File S9).

## Discussion

It is essential to employ desired materials for genetic mapping of QTLs. The present RIL showed the most abundant variations in all involved traits and thus were ideal for genetic map construction and QTL detection (Fig. 1). The present map was comprised of 26 groups and 609 loci. These valuable characteristics will be of benefit to marker transfer and the integration/comparison among different maps in peanut (Fig. 2). The genetic separation between A7 and A7^1^, B3 and B3^1^, B3^2^, and B3^3^ gave more insight into the genetic structure of peanut. The good collinearity between the present map, the physical and INT map indicated the high quality of the present linkage map (Fig. 3, Figs. S2, 3 and 4). It still needs further work to make it clear whether the insufficient number of markers or the low frequency of recombination caused the separation between A7and A7^1^, B3 to B3^1^, B3^2^ and B3^3^.

The good collinearity between the present and the physical map revealed an evolutionarily highly conserved genome sequences between diploid and tetraploid species of *Arachis* (Fig. 3). It will be significant for guiding the fine mapping and map-based cloning of QTLs by comparative mapping between the present and the physical map. The 9.82 Mb of average physical distance of the flanking markers of unique QTLs also indicated a great potential and challenge to carry out comparative mapping by using the physical map of diploid species of *Arachis* (Bertioli et al. 2016). The INT map was constructed using 16 populations and involved four diploid species *A. duranensis*, *Arachis stenosperma*, *A. ipaensis*, and *Arachis magna* and tetraploid species *A. hypogaea* (Shirasawa et al. 2013). It presented almost all the possible genetic recombination reported by all past researchers with the highest resolution of 0.7 cM/locus and the largest genetic distance of 2651 cM with 3693 loci. The high collinearity between the present map and the INT map validated the reliability of the present map and indicated a genome-wide conserved genetic make-up of tetraploid species of *Arachis* (Fig. S2). The frequent chromosome recombination between the present and the INT map might indicate an intrinsic characteristic of the present RIL populations. The comparative mapping significantly narrowed the collinear region on INT map in several groups and should be helpful to more precise genetic mapping of the present QTLs in future study.

### QTLs for yield traits

The present study revealed 92 QTLs for 11 traits. Among them, 15 QTLs were detected across three environments. Due to the poor knowledge of QTLs for peanut yield traits (Gomez Selvaraj et al. 2009; Varshney et al. 2010; Fonceka et al. 2012; Pandey et al. 2014; Huang et al. 2015; Chen et al. 2016a, b), up to 65 QTLs were newly identified. After two rounds of meta-analysis, the 70 overlapping QTLs were integrated into 33 consensus QTLs (Table S4); finally, a total of 29 pleiotropic QTLs were identified with significantly narrowed confidence interval (1.86 cM) of QTLs. The employment of meta-analysis improved the location accuracy of the QTLs and facilitated the dissection of the genetic basis of the yield traits. The present study also gave a more precise location for QTL analysis than previous researches.

For the two plant architecture traits, HMS and TBN, QTLs for both traits were located on A2, A6, B1, B6, and B9 groups with moderate phenotypic variation. The previous QTLs for the HMS were located on A3, A4, A7, B4, B7, B8, and B10 groups, and the QTLs for the TBN were located on A1 and A8 with moderate phenotypic variation (Fonceka et al. 2012; Huang et al. 2015). It indicated that the genetics basis of the HMS was dependent on peanut genotypes. It was consistent to the results in other crops that the HMS was controlled by multiple QTLs (Peiffer et al. 2014; Zhang et al. 2015). It seemed that the present QTLs for the HMS were controlled mainly by genotype because of their expression across three environments.

In the present study, nine pod and seed traits, the PL, PW, PL/PW, SL, SW, SL/SW, 100SW, 100PW, and SP were characterized. Most of them were newly identified, and the general correlations among them were observed (Table 1). The previously reported QTLs for these traits were located on A2, A3, A5, A7, A8, A9, A10, B2, B3, B5, B6, and B9 groups (Fonceka et al. 2012; Shirasawa et al. 2012; Jiang et al. 2014; Pandey et al. 2014; Huang et al. 2015, 2016; Chen et al. 2016a, b). In the present study, a total of 83 QTLs for pod and seed traits were identified, and 56 of them were newly identified; most of the identified QTLs could only explain moderate phenotypic variation. Besides the previously reported A2, A3, A5, A7, A10, and B6 groups, the QTLs were observed on B1, B4, B7, and B8 for the first time. It seemed that the loci controlling the yield-determining traits distributed all over the whole genome, which also indicated a complex genetic basis of the yield-determining traits. It also was practicable to clone the QTLs with moderate phenotypic variation for seed size and weight in peanut as well as in soybean (Xie et al. 2014).

In the present study, the 100PW and 100SW was more than twofold for Fuchuan Dahuasheng than those for ICG6375, and the measurement of 64.3 g for 100PW and 25.5 g for 100SW of ICG6375 were almost the lowest level of these two traits in cultivated peanut (Table S1). The identified QTLs for yield traits were contributed by Fuchuan Dahuasheng with additive effect and moderate phenotypic variation explained, no QTLs for 100PW and 100SW was observed from ICG6375; the question was whether the genetic factors of these two traits could not be further dissected in ICG6375. It could be deduced that the present QTLs for pod- and seed-related traits might be the part of the most fundamental factors contributing to the yield traits in peanut.

The QTL *qPLA7.1b*, *qPLB1.1a*, *qSLB1.1a*, *qSWB1.1a*, and *qSL/SWA2.1a* should be candidate for MAS and map-based cloning in further study for their more than 10 % of phenotypic variation. For the PL, only the *qPLA7.1c* was detected across all environments, it seemed that the PL was controlled by intensive interaction between genotype and environments. Interestingly, the *qPLA5.1a* was overlapped with the QTL for maturity by common marker PM45 associated with pod maturity, and the correlation was not observed between the PL and the maturity (Gomez Selvaraj et al. 2009). It was a common sense that the completion of pod expanding often symbolized the maturity of pod during normal peanut ontogenesis. Further study is still needed to clarify whether the same one QTL contributed to both the maturity and the PL in present RIL population. In *B. napus*, a similar trait, the silique length was also controlled by multiple QTLs (Shi et al. 2009; Yang et al. 2012; Li et al. 2014). It was expected that more QTLs of PL should be identified using different peanut genotypes. The QTLs of seed size were well characterized in soybean. The seed size-related QTLs were distributed over 16 chromosomes. Many of them accounted minor or moderate phenotypic variation, indicating a comprehensive genetic basis of seed size (Salas et al. 2006; Xu et al. 2011; Niu et al. 2013). Because of the close genetic relationship between soybean and peanut (Shirasawa et al. 2013), it is possible that multiple QTLs actually contributed to pod and seed size in peanut. And up to date, more than ten genes associated with grain shape and size have been characterized well in rice, and these genes acted in independent genetic pathways (Zuo and Li 2014; Wang et al. 2015b). More interestingly, the QTLs *GS5, qSW/GW* and *qGW7* controlled seed size and affected the grain quality in rice (Peng et al. 2015; Wang et al. 2015b). It still needs further study on whether these QTLs for seed traits affected quality traits in peanut as well as *GS5* and *qSW/GW* in rice.

For the two yield-directly related traits 100PW and 100SW, all QTLs were newly identified. The QTLs *q100SWA7.1c* and *q100PWB8.1a* could be further studied for their more than 10 % of phenotypic variation. And the present 100PW and 100SW were possibly controlled mainly by genotype because the *q100SWA7.1a*, *q100SWB6.1a*, *q100SWb8.1a*, *q100PWA7.1a*, *q100SWA7.1a*, and *q100SWB6.1b* were expressed across the three environments. The 100PW and 100SW were controlled by multiple QTLs in soybean (Han et al. 2012; Kato et al. 2014; Xie et al. 2014) and *B. napus* (Li et al. 2014; Liu et al. 2015). In the present study, the significant correlation was observed between 100PW and 100SW, and the QTLs controlling both of them were often co-localized on A7 and B8 groups. However, the QTLs for 100PW were also located on A5, A6, and B7 groups; meanwhile, the QTLs for 100SW were located on A2 and B6 groups. The unanswered question was whether the shell contributed to the different locations of QTLs for 100PW and 100SW?

### The co-localization of the QTLs for yield-related traits

As the results of meat analysis, the significant co-localization of QTLs was observed, and 29 pleiotropic QTLs were identified. The present pleiotropic QTLs should contribute to better understanding of yield components and the linked markers will facilitate MAS breeding in peanut. The genetic bases of yield component had been studied, and the pod- and seed-related traits were highly valued in peanut (Gomez Selvaraj et al. 2009; Shirasawa et al. 2012; Jiang et al. 2014; Pandey et al. 2014; Huang et al. 2015), soybean(Xu et al. 2011; Niu et al. 2013; Kato et al. 2014; Nemli et al. 2014; Xie et al. 2014), rapeseed (Chen et al. 2007; Li et al. 2014, 2015; Liu et al. 2015; Shi et al. 2009, 2015), and rice (Zuo and Li 2014; Wang et al. 2015b). The significant co-localizations indicated that the yield traits were dependent on each other; the pleiotropic QTLs were the important genetic factors which contributed to the yield traits in the present RIL population. It means that the locus of pleiotropic QTL containing multiple, tightly linked, trait-specific genes or the genes that affect multiple traits (Hall et al. 2006). It is often observed that the QTLs and genes acted as affecting yield exhibiting pleiotropic effects on more than one trait (Shi et al. 2009). In soybean, it was the pleiotropy contributed to the seed size and weight, the QTL X40, X53, X83–X85, and X92 simultaneously controlled two of the seed length, seed width, and 100-seed weight (Xie et al. 2014). In *Brassina napus*, the pleiotropic QTLs were also identified. For example, the QTLs *uq. A09–1* and *uq. A09–3* contributed to both the seed weight and the silique length (Li et al. 2014), the gene *ARF18* simultaneously affected the seed weight and silique length (Liu et al. 2015). And in rice, the QTLs contributed to the seed size and weight were reported (Zuo and Li 2014; Wang et al. 2015b). It could be reasoned that the significant pleiotropic QTLs might be resulted from the artificial selection in long term of peanut breeding, because the yield-determining QTLs were all from Fuchuan Dahuasheng. The QTL cluster was also a mechanism for rapid multiple traits evolution (Yoshizawa et al. 2013).

## Electronic supplementary material

Fig. S1 (JPEG 2585 kb)

Fig. S2 (JPEG 903 kb)

Fig. S3 (JPEG 584 kb)

Fig. S4 (JPEG 1125 kb)

Fig. S5 (JPEG 173 kb)

ESM 6 (XLSX 52 kb)

ESM 7 (XLSX 28.9 kb)

ESM 8 (XLSX 293 kb)

ESM 9 (XLSX 41 kb)

ESM 10 (XLSX 22.6 kb)

ESM 11 (XLSX 20.8 kb)

ESM 12 (XLSX 11.1 kb)

ESM 13 (XLSX 13.6 kb)

ESM 14 (XLSX 11.8 kb)

ESM 15 (DOCX 17.7 kb)

ESM 16 (DOCX 18.2 kb)

ESM 17 (DOCX 20.8 kb)

ESM 18 (DOCX 24.2 kb)
