# Supplementary figures and images for: Genetic mapping of yield traits using RIL population derived from Fuchuan Dahuasheng and ICG6375 of peanut (Arachis hypogaea L.)
Source: Mol Breed. 2017 Jan 30;37(2):17. doi: 10.1007/s11032-016-0587-3 (PMC5285419; doi:10.1007/s11032-016-0587-3)

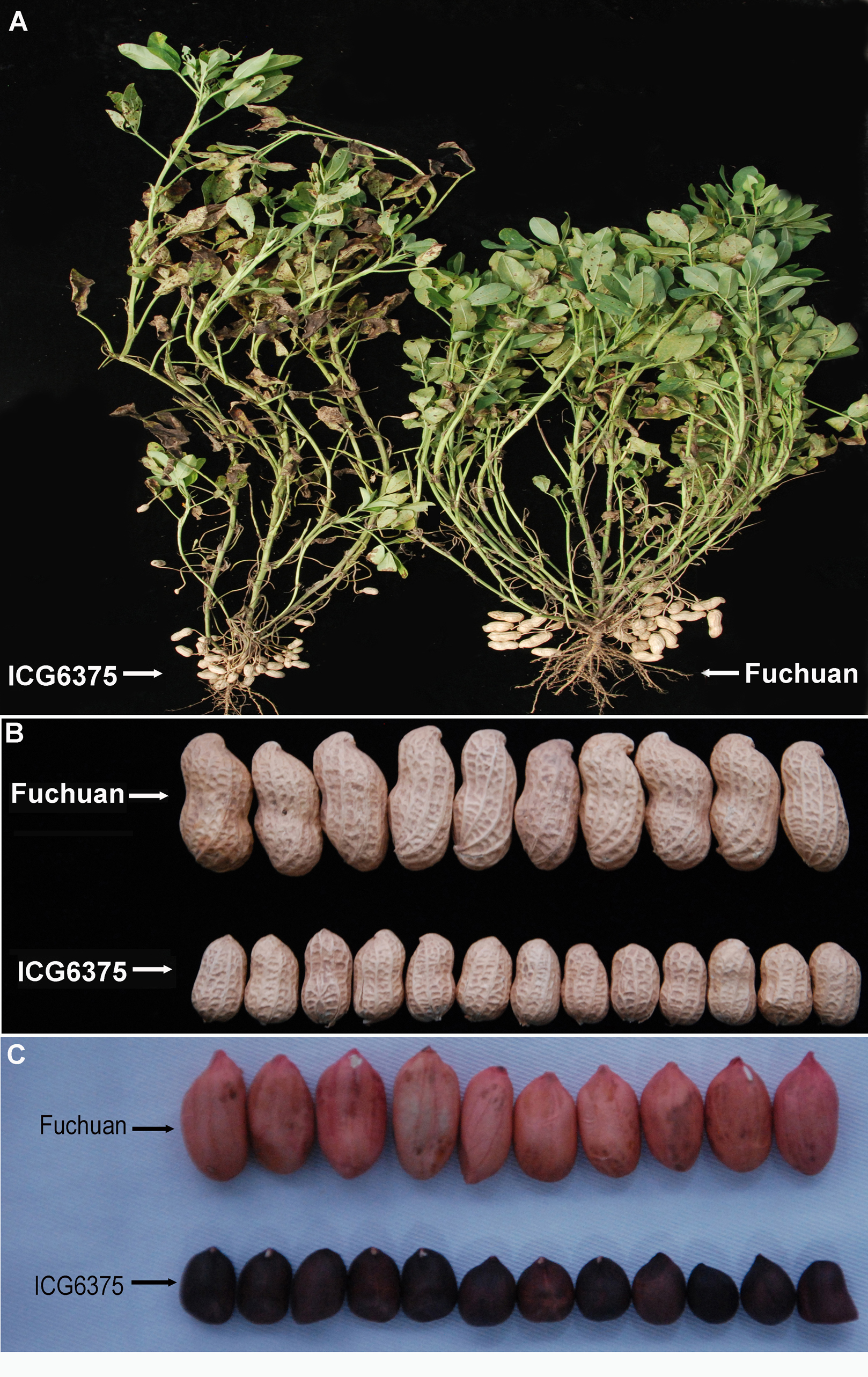

Supplement: Supplementary file 1 — (JPEG 2585 kb) [file 11032_2016_587_MOESM1_ESM.jpg]

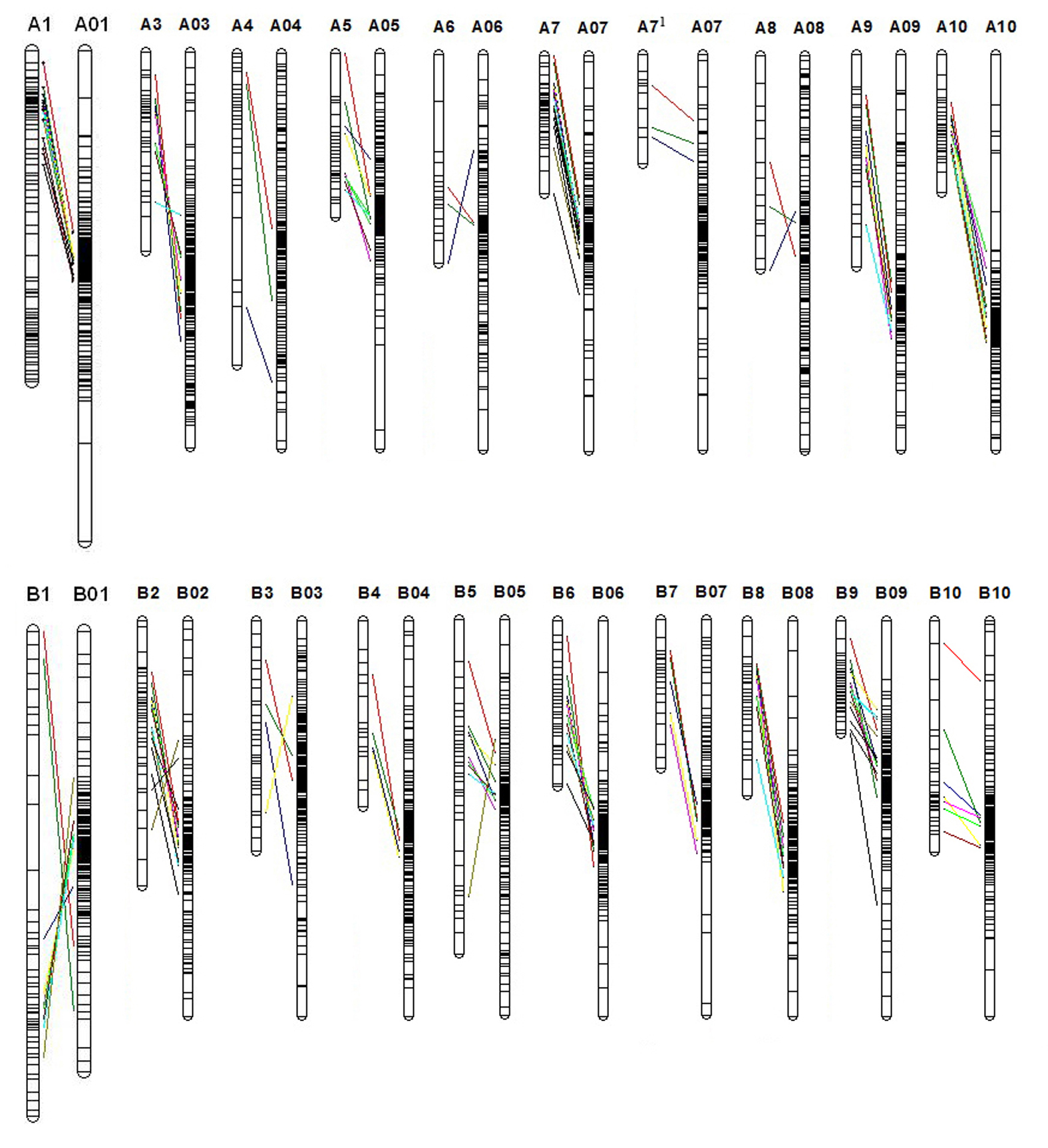

Supplement: Supplementary file 2 — (JPEG 903 kb) [file 11032_2016_587_MOESM2_ESM.jpg]

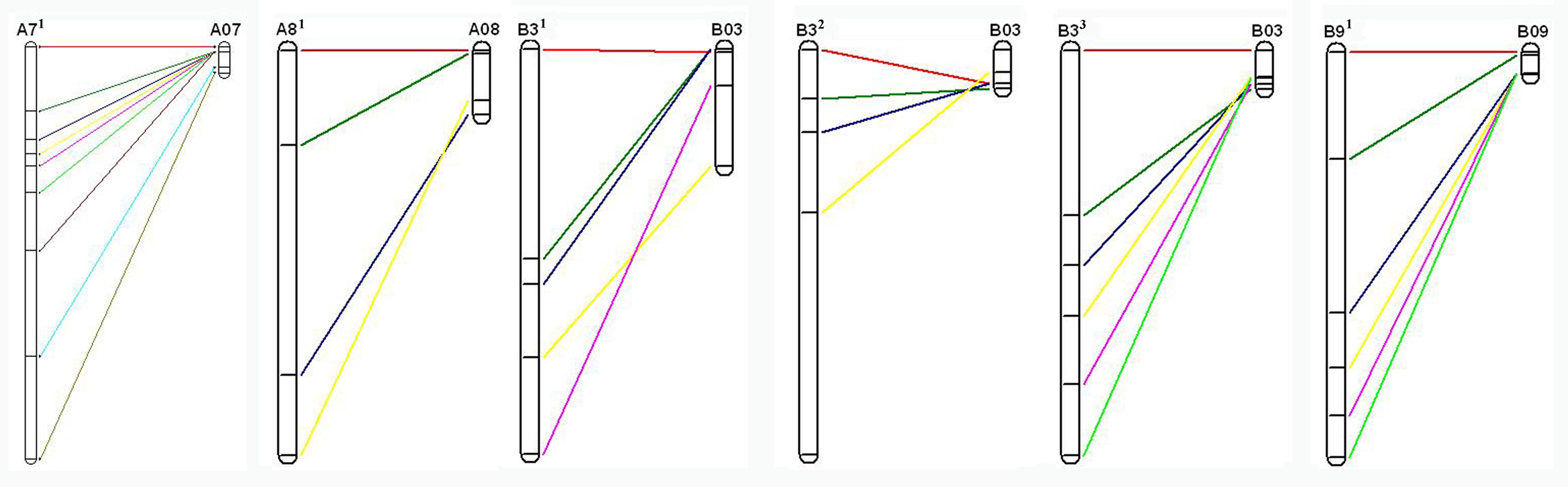

Supplement: Supplementary file 3 — (JPEG 584 kb) [file 11032_2016_587_MOESM3_ESM.jpg]

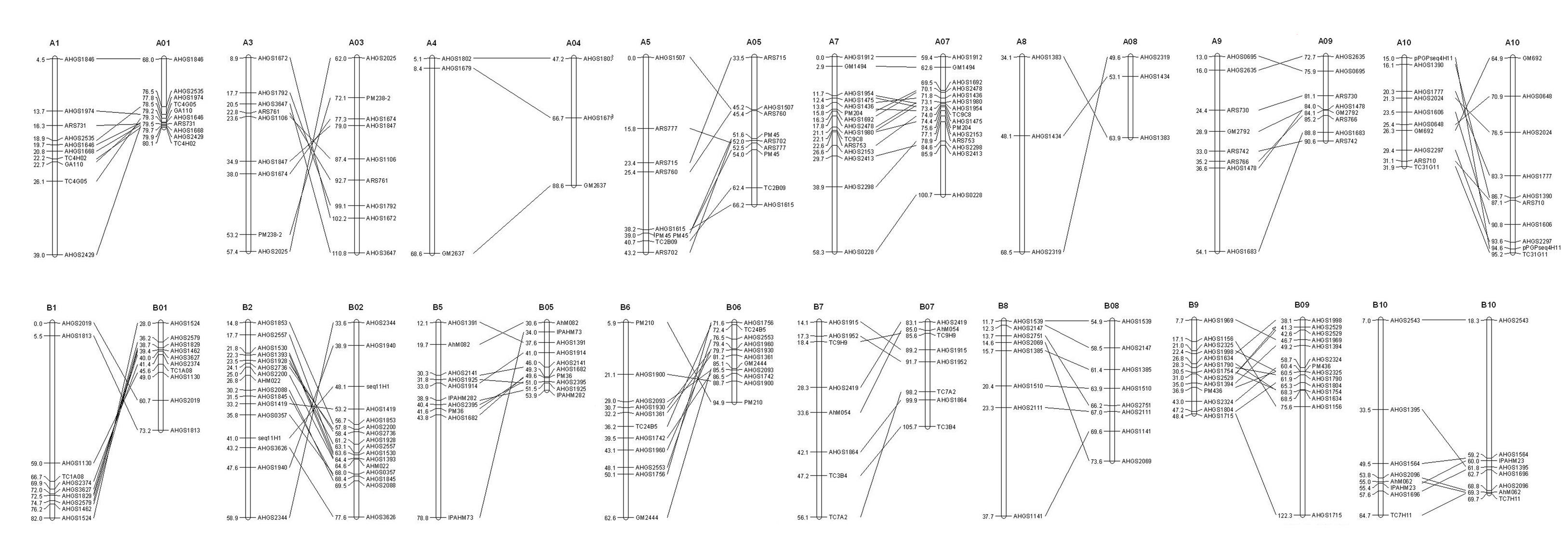

Supplement: Supplementary file 4 — (JPEG 1125 kb) [file 11032_2016_587_MOESM4_ESM.jpg]

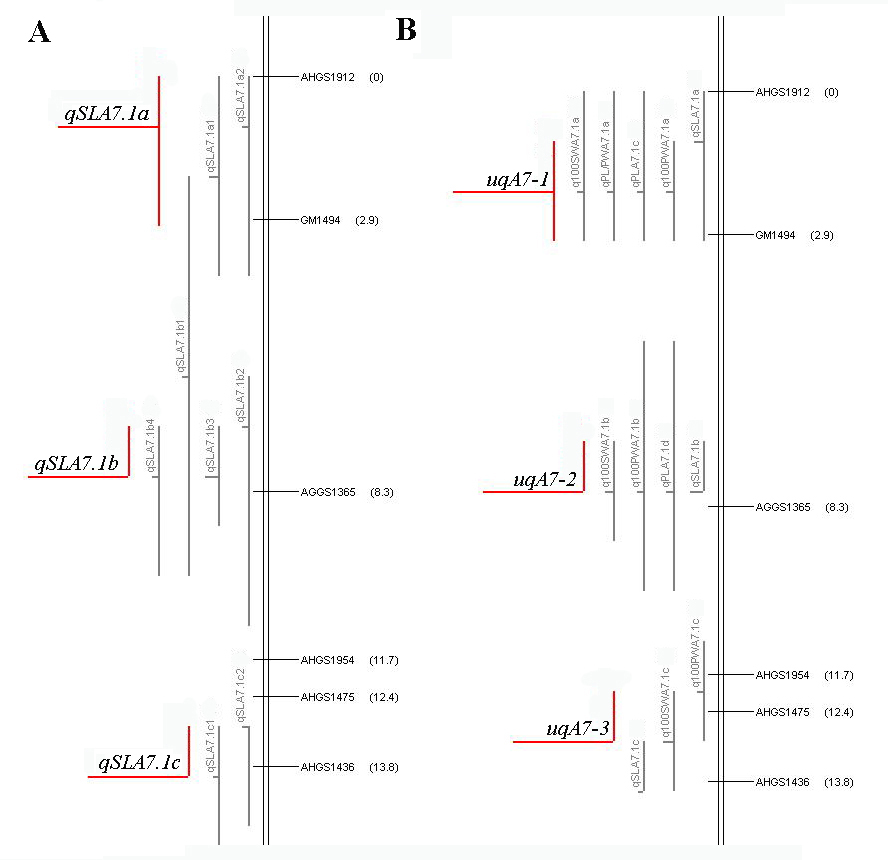

Supplement: Supplementary file 5 — (JPEG 173 kb) [file 11032_2016_587_MOESM5_ESM.jpg]
