# Supplementary material for: Genetic mapping of yield traits using RIL population derived from Fuchuan Dahuasheng and ICG6375 of peanut (Arachis hypogaea L.)
Source: Mol Breed. 2017 Jan 30;37(2):17. doi: 10.1007/s11032-016-0587-3 (PMC5285419; doi:10.1007/s11032-016-0587-3)
Supplement: Supplementary file 15 — (DOCX 17.7 kb) [file 11032_2016_587_MOESM15_ESM.docx]

Supplied Table 1: Significance analysis of the eleven traits between Fuchuan Dahuasheng and ICG6375 using averaged value across three environments.

| Trait | HMS | TBN | PL | PW | PL/PW | SL |
| --- | --- | --- | --- | --- | --- | --- |
| Fuchun | \| 50.6±3.1 \| \| --- \| | 16.3±2.2 | 3.19±0.48 | 1.3±0.19 | 2.2±0.1 | 1.53±0.9 |
| ICG6375 | \| 85.3±.9 \| \| --- \| | 11.2±2.19 | 1.96±0.1.3 | 0.89±1.3 | 1.9±0.1 | 1.02±0.2 |
| F-value | 24.8** | 132.4** | 26.4** | 52.3** | 32.6** | 42.5** |
| H^2^ | 0.40 | 0.80 | 0.87 | 0.87 | 0.88 | 0.85 |
| Trait | SW | SL/SW | 100PW | 100SW | SP |  |
| Fuchuan | 0.81±0.3 | 1.92±0.1 | 168.7±24.8 | 52.9±4.7 | 0.69±0.1 |  |
| ICG6375 | 0.73±0.1 | 1.3±0.1 | 64.3±5.9 | 25.5±2.5 | 0.76±0.5 |  |
| F-value | 6.2 | 60.3** | 24.8** | 39.1** | 2.54 |  |
| H^2^ | 0.95 | 0.80 | 0.90 | 0.75 | 0.59 |  |

PL pod length, PW pod width, PL/PW pod length to width, SL seed length, SW seed width, SL/SW seed length to seed width,100PW 100 pods weight, 100SW 100 seeds weight, SP shelling percentage. H^2^, broad-sense heritability on entry-mean basis; **Significant at *P* <0.01.
