# Supplementary material for: Genetic mapping of yield traits using RIL population derived from Fuchuan Dahuasheng and ICG6375 of peanut (Arachis hypogaea L.)
Source: Mol Breed. 2017 Jan 30;37(2):17. doi: 10.1007/s11032-016-0587-3 (PMC5285419; doi:10.1007/s11032-016-0587-3)
Supplement: Supplementary file 16 — (DOCX 18.2 kb) [file 11032_2016_587_MOESM16_ESM.docx]

| LGs on FI maps | Collinear Genetic distance on FI maps (cM) | LGs on INT maps | Collinear genetic distance on INT maps(cM) | Number of common marker |
| --- | --- | --- | --- | --- |
| A1 | 34.49 | A01 | 12.09 | 10 |
| A3 | 48.57 | A03 | 48.79 | 9 |
| A4 | 63.49 | A04 | 41.38 | 3 |
| A5 | 43.18 | A05 | 32.72 | 8 |
| A6 | 26.39 | A06 | 25.78 | 3 |
| A7 | 58.25 | A07 | 41.31 | 15 |
| A7^1^ | 21.25 | A07 | 17.51 | 3 |
| A8 | 34.45 | A08 | 14.36 | 3 |
| A9 | 41.04 | A09 | 17.95 | 8 |
| A10 | 16.89 | A10 | 30.25 | 10 |
| B1 | 82.01 | B01 | 45.18 | 10 |
| B2 | 44.10 | B02 | 43.95 | 16 |
| B3 | 55.36 | B03 | 69.09 | 4 |
| B3^2^ | 19.71 | B03 | 7.29 | 3 |
| B4 | 25.42 | B04 | 27.60 | 4 |
| B5 | 66.76 | B05 | 23.37 | 10 |
| B6 | 56.63 | B06 | 23.24 | 10 |
| B7 | 42.04 | B07 | 22.67 | 8 |
| B8 | 26.20 | B08 | 34.86 | 8 |
| B9 | 40.70 | B09 | 84.16 | 14 |
| B10 | 57.70 | B10 | 51.41 | 8 |
| Total | 904.63 |  | 714.96 | 167 |

Supplementary Table S2. The comparison between FI map and the INT map based on common markers, the genetic distance of collinear region of corresponded linkage group were showed.
