# Supplementary material for: Genetic mapping of yield traits using RIL population derived from Fuchuan Dahuasheng and ICG6375 of peanut (Arachis hypogaea L.)
Source: Mol Breed. 2017 Jan 30;37(2):17. doi: 10.1007/s11032-016-0587-3 (PMC5285419; doi:10.1007/s11032-016-0587-3)
Supplement: Supplementary file 17 — (DOCX 20.8 kb) [file 11032_2016_587_MOESM17_ESM.docx]

Table S3 Unique QTLs based on meta-analysis

| Unique QTL | LG | P | CI | QTLs integrated | | | | |
| --- | --- | --- | --- | --- | --- | --- | --- | --- |
| *uqA2-1* | A2 | 37.91 | 36.77-39.05 | *qPL/PWA2.1a* | *qSL/SWA2.1a* |  |  |  |
| *uqA2-2* | A2 | 47.23 | 45.38-49.08 | *qHMSA2.1a* | *qPL/PWA2.1b* | *qSL/SWA2.1b* |  |  |
| *uqA2-2* | A2 | 59.78 | 58.44-61.13 | *qPWA2.1a* | *q100SWA2.1a* |  |  |  |
| *uqA3-1* | A3 | 49.21 | 48.71-49.71 | *qPLA3.1a* | *qPL/PWA3.1a* | *qSL/SWA3.1a* |  |  |
| *uqA3-2* | A3 | 61.41 | 59.9-62.4 | *qPLA3.1b* | *qSWA3.1a* |  |  |  |
| *uqA5-1* | A5 | 35.81 | 36.82-37.6 | *qPLA5.1a^C^* | *qPWA5.1a* | *qPL/PWA5.1a* | *qSLA5.1a* | *q100PWA5.1a^C^* |
| *uqA5-2* | A5 | 43.74 | 434-4.48 | *qPLA5.1b^C^* | *qPWA5.1b* | *qPL/PWA5.1b* | *qSLA5.1b* | *q100PWA5.1c* |
| *uqA7-1* | A7 | 2.46 | 1.85-3.06 | *qPLA7.1a* | *qPL/PWA7.1a* | *qSLA7.1a^C^* | *q100PWA7.1a^C^* | *q100SWA7.1a* |
| *uqA7-2* | A7 | 8.24 | 7.68-8.8 | *qPLA7.1b* | *qSLA7.1b^C^* | *q100PWA7.1b* | *q100SWA7.1b^C^* | |
| *uqA7-3* | A7 | 13.44 | 12.95-13.93 | *qSLA7.1c^C^* | *q100PWA7.1c* | *q100SWA7.1c^C^* |  |  |
| *uqA7-4* | A7 | 26.05 | 25.5-26.6 | *qPLA7.1c^C^* | *qSL/SWA7.1a^C^* |  |  |  |
| *uqA7-5* | A7 | 35.21 | 34.29-36.14 | *qPLA7.1d^C^* | *qPL/PWA7.1b* | *qSL/SWA7.1b^C^* |  |  |
| *uqA10-1* | A10 | 14.21 | 13.14-15.28 | *qPL/PWA10.1a* | *qSLA10.1a* |  |  |  |
| *uqA10-2* | A10 | 17.5 | 16.9-18.09 | *qSLA10.1a* | *qSWA10.1a^C^* |  |  |  |
| *uqA10-3* | A10 | 24.88 | 24.6-25.15 | *qPL/PWA10.1b* | *qSWA10.1b^C^* |  |  |  |
| *uqB1-1* | B1 | 47.47 | 45.41-49.54 | *qPLB1.1a* | *qPWB1.1a* | *qPL/PWB1.1a* | *qSLB1.1a* | *qSPB1.1a* |
| *uqB1-2* | B1 | 66.5 | 65.73-67.27 | *qPLB1.1b* | *qPL/PWB1.1c* |  |  |  |
| *uqB1-3* | B1 | 72.36 | 71.94-72.77 | *qPL/PWB1.1d* | *qSWB1.1b* |  |  |  |
| *uqB2-1* | B2 | 41.01 | 40.29-41.73 | *qSL/SWB2.1a* | *qSWB2.1a^C^* |  |  |  |
| *uqB4-1* | B4 | 43.3 | 42.77-43.82 | *qPLB4.1a* | *qSLB4.1a^C^* | *qSL/SWB4.1a* | *q100SWB4.1a* |  |
| *uqB6-1* | B6 | 11.95 | 9.48-14.42 | *qSLB6.1a* | *qSL/SWB6.1a* |  |  |  |
| *uqB6-2* | B6 | 32.64 | 32.12-33.15 | *qHMSB6.1a^C^* | *qSLB6.1b^C^* | *qTBNB6.1a* |  |  |
| *uqB6-3* | B6 | 42.3 | 41.63-42.97 | *qHMSB6.1b^C^* | *qSWB6.1a* | *q100SWB6.1a^C^* |  |  |
| *uqB6-4* | B6 | 47.66 | 47.02-48.3 | *qHMSB6.1c^C^* | *qSWB6.1b* | *q100SWB6.1b* | *qSPB6.1a^C^* |  |
| *uqB6-5* | B6 | 55.61 | 53.87-57.35 | *qSWB6.1c* | *q100SWB6.1c^C^* |  |  |  |
| *uqB7-1* | B7 | 14.11 | 12.48-15.74 | *qSLB7.1a^C^* | *q100PWB7.1a* |  |  |  |
| *uqB7-2* | B7 | 46.57 | 44.99-48.15 | *qPL/PWB7.1a^C^* | *qSL/SWB7.1c* |  |  |  |
| *uqB8-1* | B8 | 26.71 | 26.29-27.31 | *qPLB8.1a* | *qPWB8.1a^C^* | *q100PWB8.1a^C^* | *q100SWB8.1a^C^* | |
| *uqB8-2* | B8 | 35.91 | 35.45-36.37 | *qPWB8.1b^C^* | *q100PWB8.1b* |  |  |  |

LG linkage group, P position, CI confidence interval, ^C^Consensus QTL
