# Supplementary material for: Genetic mapping of yield traits using RIL population derived from Fuchuan Dahuasheng and ICG6375 of peanut (Arachis hypogaea L.)
Source: Mol Breed. 2017 Jan 30;37(2):17. doi: 10.1007/s11032-016-0587-3 (PMC5285419; doi:10.1007/s11032-016-0587-3)
Supplement: Supplementary file 18 — (DOCX 24.2 kb) [file 11032_2016_587_MOESM18_ESM.docx]

Supplementary Table S4. The detailed QTL information where were integrated into consensus QTLs

| Trait | LG | Consensus QTL | Position | CI | P | LOD | AE | R2 | CI | En |
| --- | --- | --- | --- | --- | --- | --- | --- | --- | --- | --- |
| HMS | A6 | *qHMSA6.1b* | 63.61 | 61.7-67.8 | 63.61 | 4.640608 | -3.7804 | 9.0768 | 61.7-67.8 | E1 |
| HMS | A6 |  |  |  | 63.61 | 3.127036 | -3.5947 | 6.305 | 62.6-67.4 | E3 |
| HMS | B6 | *qHMSB6.1c* | 47.66 | 47.02-48.3 | 47.41 | 2.933768 | -3.0877 | 5.9464 | 47.1-48.7 | E1 |
| HMS | B6 |  |  |  | 48.11 | 5.294245 | -4.265 | 8.9007 | 45.3-48.6 | E3 |
| HMS | B6 |  |  |  | 48.11 | 4.332248 | -3.6504 | 7.6873 | 45.8-48.6 | PooledE3 |
| HMS | B6 | *qHMSB6.1a* | 30.44 | 29.89-30.98 | 30.81 | 3.550489 | -3.7294 | 6.7244 | 30.3-31.8 | E3 |
| HMS | B6 |  |  |  | 30.01 | 4.896851 | -4.0285 | 9.245 | 29.8-31.4 | PooledE3 |
| HMS | B6 | *qHMSB6.1b* | 41.98 | 41.19-42.77 | 41.61 | 5.457112 | -4.3226 | 9.1425 | 40.6-42.6 | E3 |
| HMS | B6 |  |  |  | 42.61 | 4.04126 | -3.5424 | 7.4758 | 40.5-43.1 | PooledE3 |
| PL | A7 | *qPLA7.1a* | 26.05 | 25.5-26.6 | 26.61 | 3.109663 | 0.0836 | 6.5632 | 25.6-27.1 | E1 |
| PL | A7 |  |  |  | 25.41 | 2.977199 | 0.1239 | 6.4934 | 24.1-25.7 | E3 |
| PL | A7 | *qPLA7.1b* | 35.6 | 34.51-36.69 | 35.21 | 4.416938 | 0.1006 | 9.6533 | 34.1-37 | E1 |
| PL | A7 |  |  |  | 35.21 | 2.959826 | 0.1251 | 6.6515 | 33.5-37 | E3 |
| PL | A5 | *qPLA5.1a* | 37.82 | 36.85-38.8 | 38.21 | 2.65798 | 0.1195 | 5.7688 | 37-39.7 | E3 |
| PL | A5 |  |  |  | 37.41 | 4.914224 | 0.1447 | 10.5456 | 35.8-38.6 | E3 |
| PL | A5 | *qPLA5.1b* | 43.81 | 42.78-44.84 | 42.71 | 4.903366 | 0.1475 | 10.9215 | 40.7-44.7 | E3 |
| PL | A5 |  |  |  | 44.21 | 3.648208 | 0.1295 | 8.8505 | 43-45.4 | E1 |
| PW | B8 | *qPWB8.1a* | 26.71 | 26.2-28.2 | 26.71 | 3.543974 | 0.0394 | 7.2574 | 26.2-28.2 | E1 |
| PW | B8 |  |  |  | 26.71 | 3.821933 | 0.0616 | 8.5647 | 25.7-28 | E3 |
| PW | B8 |  |  |  | 26.71 | 4.173724 | 0.0578 | 9.385 | 26.2-29 | PooledE3 |
| PW | B8 | *qPWB8.1b* | 35.91 | 35.27-36.55 | 35.91 | 3.266015 | 0.0606 | 8.4774 | 34.7-36.2 | PooledE3 |
| PW | B8 |  |  |  | 35.91 | 3.161781 | 0.0467 | 8.0978 | 33.9-36.3 | E1 |
| PL/PW | B7 | *qPL/PWB7.1a* | 46.4 | 44.6-48.21 | 47.11 | 6.178067 | 0.0886 | 6.0614 | 44.4-52.3 | E1 |
| PL/PW | B7 |  |  |  | 47.11 | 3.003257 | 0.0693 | 7.059 | 43.7-56.1 | E3 |
| PL/PW | B7 |  |  |  | 46.11 | 3.246471 | 0.0617 | 7.0602 | 42.9-47.2 | E3 |
| SL | B4 | *qSLB4.1a* | 43.18 | 42.1-44.27 | 43.51 | 2.807818 | 0.0393 | 5.4425 | 39.8-45.2 | E1 |
| SL | B4 |  |  |  | 43.11 | 3.337676 | 0.0561 | 7.814 | 42.1-44.5 | PooledE3 |
| SL | A7 | *qSLA7.1b* | 8.18 | 7.43-8.93 | 6.61 | 2.885993 | 0.0432 | 6.2993 | 2.8-10.7 | E1 |
| SL | A7 |  |  |  | 7.61 | 2.660152 | 0.0417 | 6.037 | 6.5-11.2 | E3 |
| SL | A7 |  |  |  | 8.31 | 7.024973 | 0.0626 | 13.6087 | 7.6-9.5 | E3 |
| SL | A7 |  |  |  | 8.31 | 2.944625 | 0.0492 | 6.575 | 7.6-10.7 | PooledE3 |
| SL | A7 | *qSLA7.1c* | 14.13 | 13.31-14.96 | 14.81 | 2.577633 | 0.0413 | 5.7573 | 13.5-16.4 | E1 |
| SL | A7 |  |  |  | 13.81 | 3.17481 | 0.0385 | 6.7082 | 13.1-15.1 | E2 |
| SL | A7 | *qSLA7.1a* | 1.51 | 0.12-3.14 | 2.01 | 4.245385 | 0.0524 | 9.6506 | 0-4.6 | E3 |
| SL | A7 |  |  |  | 1.01 | 2.508143 | 0.0476 | 6.1471 | 0-4.6 | PooledE3 |
| SL | B7 | *qSLB7.1a* | 14.11 | 12-16.4 | 14.11 | 2.542888 | 0.0404 | 5.6421 | 11.21-7.3 | E3 |
| SL | B7 |  |  |  | 14.11 | 3.385451 | 0.0428 | 6.4852 | 12-16.4 | E3 |
| SL | B7 | *qSLB7.1b* | 20.41 | 19.14-21.68 | 20.41 | 3.216069 | 0.0452 | 7.0845 | 19.6-22.8 | E3 |
| SL | B7 |  |  |  | 20.41 | 3.513572 | 0.0442 | 6.8995 | 18.5-22.7 | E3 |
| SL | B6 | *qSLB6.1a* | 32.31 | 31.68-32.94 | 32.31 | 2.701412 | 0.0413 | 5.5022 | 30.1-35.4 | E1 |
| SL | B6 |  |  |  | 32.31 | 2.72747 | 0.0489 | 6.2298 | 31.8-33.1 | PooledE3 |
| SW | B2 | *qSWB2.1a* | 41.01 | 40.5-42.5 | 41.01 | 3.270358 | -0.0223 | 7.0747 | 40.5-42.5 | E3 |
| SW | B2 |  |  |  | 41.01 | 2.9924 | -0.0201 | 6.0637 | 40.5-42.5 | E3 |
| SW | A10 | *qSWA10.1a* | 17.68 | 16.96-18.4 | 14.21 | 4.260586 | 0.0276 | 8.6667 | 12.41-7.1 | E1 |
| SW | A10 |  |  |  | 16.01 | 2.729642 | 0.026 | 5.7699 | 14.6-17.1 | PooledE3 |
| SW | A10 | *qSWA10.1b* | 24.88 | 24.63-25.15 | 24.91 | 4.649294 | 0.0288 | 9.4175 | 24.4-25 | E1 |
| SW | A10 |  |  |  | 24.91 | 4.581976 | 0.0327 | 10.4215 | 23.8-25.3 | PooledE3 |
| SL/SW | A7 | *qSL/SWA7.1a* | 25.41 | 24.2-26.3 | 23.61 | 2.931596 | 0.0425 | 5.9593 | 23.2-24.4 | E3 |
| SL/SW | A7 |  |  |  | 25.41 | 3.65038 | 0.0467 | 7.423 | 24.2-26.3 | E3 |
| SL/SW | A7 | *qSL/SWA7.1b* | 34.47 | 33.65-35.29 | 34.21 | 3.32899 | 0.0445 | 6.6528 | 33.3-35.2 | E3 |
| SL/SW | A7 |  |  |  | 35.21 | 2.988056 | 0.0539 | 6.4623 | 33.4-36.6 | E1 |
| 100PW | A7 | *q100PWA7.1a* | 2.46 | 1.85-3.06 | 2.91 | 2.673181 | 7.8386 | 5.968 | 2-3.9 | PooledE1+2 |
| 100PW | A7 |  |  |  | 1.01 | 3.266015 | 10.2232 | 7.7852 | 0-3.5 | PooledE3 |
| 100PW | B8 | *q100PWB8.1a* | 26.71 | 26.4-27.5 | 26.71 | 3.960912 | 10.4664 | 8.4528 | 26.3-27.7 | E3 |
| 100PW | B8 |  |  |  | 26.71 | 4.994571 | 12.341 | 11.2019 | 26.2-28.7 | PooledE3 |
| 100PW | A5 | *q100PWA5.1a* | 35.91 | 34.9-36.2 | 35.81 | 3.090119 | 9.5158 | 6.7867 | 35.1-38 | E3 |
| 100PW | A5 |  |  |  | 35.81 | 2.9924 | 9.5956 | 6.7322 | 35-37.4 | PooledE3 |
| 100SW | A7 | *q100SWA7.1c* | 13.53 | 12.74-14.32 | 13.81 | 2.859935 | 2.4225 | 5.8745 | 12.9-15.8 | E1 |
| 100SW | A7 |  |  |  | 13.41 | 4.032573 | 2.1274 | 10.2484 | 12.4-14.3 | E2 |
| 100SW | A7 | *q100SWA7.1b* | 8.31 | 7.36-9.26 | 8.31 | 3.029316 | 2.7703 | 5.9737 | 7.5-10.1 | E3 |
| 100SW | A7 |  |  |  | 8.31 | 4.204126 | 2.3931 | 8.9899 | 7.4-10.2 | PooledE1+2 |
| 100SW | B8 | *q100SWB8.1a* | 26.71 | 25.91-27.51 | 26.71 | 4.445168 | 3.5799 | 9.8103 | 26.6-28.6 | E3 |
| 100SW | B8 |  |  |  | 26.71 | 3.37025 | 3.2753 | 7.9033 | 26-28.7 | PooledE3 |
| 100SW | B6 | *q100SWB6.1a* | 43.11 | 42.6-43.6 | 43.11 | 3.963084 | 2.8743 | 8.0239 | 42.6-43.8 | E1 |
| 100SW | B6 |  |  |  | 43.11 | 2.892508 | 1.9819 | 6.1512 | 42.3-44.1 | PooledE1+2 |
| 100SW | B6 | *q100SWB6.1c* | 55.61 | 53.53-57.69 | 55.61 | 2.801303 | 2.6935 | 5.2376 | 55.1-60.4 | E3 |
| 100SW | B6 |  |  |  | 55.61 | 3.39848 | 2.1498 | 7.1668 | 54.6-61.3 | PooledE1+2 |
| SP | B6 | *qSPB6.1a* | 50.04 | 49.45-50.64 | 49.71 | 3.131379 | 3.3283 | 7.6044 | 48.3-51.2 | PooledE1+2 |
| SP | B6 |  |  |  | 50.11 | 2.85342 | 2.5314 | 6.8645 | 49.35-0.6 | PooledE3 |
| SP | A10 | *qSPA10.1a* | 34.75 | 34.13-35.37 | 33.71 | 2.714441 | 2.4143 | 5.5748 | 32.9-34.6 | E1 |
| SP | A10 |  |  |  | 35.91 | 3.103149 | 1.9372 | 7.2373 | 35.3-37.1 | E2 |

LG linkage group, P position (cM), CI confidence interval at P = 0.05, AE additive effect, En environment
